# Gastrointestinal parasites in an isolated Norwegian population of wild red deer (*Cervus elaphus*)

**DOI:** 10.1186/s13028-014-0059-x

**Published:** 2014-10-08

**Authors:** Rebecca K Davidson, Susan J Kutz, Knut Madslien, Eric Hoberg, Kjell Handeland

**Affiliations:** Norwegian Veterinary Institute, Pb 750 Sentrum, NO-0106 Oslo, Norway; Department of Ecosystem and Public Health, Faculty of Veterinary Medicine, University of Calgary, 3330 Hospital Drive NW, Calgary, Alberta T2N 4 N1 Canada; Canadian Cooperative Wildlife Health Centre-Alberta Node, Faculty of Veterinary, Medicine, University of Calgary, 3330 Hospital Drive NW, Calgary, Alberta T2N 4 N1 Canada; US National Parasite Collection, USDA, Agricultural Research Service, BARC East No. 1180, Beltsville, MD 20705 USA; Current address: HD-diagnostikk AS, Kalkbrennerveien 12, NO-1487 Hakadal, Norway

**Keywords:** Abomasum, Caecum, Diversity, *Cervus elaphus*, *Ostertagia*, Red deer, small intestine, *Spiculopteragia*

## Abstract

**Background:**

Thirteen red deer (*Cervus elaphus*), culled from the isolated population at the Mongstad Oil Refinery, Norway, were investigated for gastrointestinal helminths. These animals, enclosed by the refinery fence, do not have contact with other ruminants and have a high population density considering the available browsing area (1 km^2^) within the refinery site (3 km^2^). The population was estimated to be 110-130 at the time of culling.

**Results:**

The helminth fauna among these sampled red deer was enumerated and species were identified based on morphology. *Ostertagia leptospicularis/O. kolchida* was detected in 83% [CI 55 - 95%], *Spiculopteragia spiculoptera/S. mathevossiani* in 92% [CI 65 - 99%] and *Trichostrongylus axei* in 42%, [CI 19 - 68%] of the abomasa examined. Characterisation of the intestinal parasite fauna revealed *Capillaria bovis, Cooperia oncophora*, *Oesophagostomum venulosum*, *Trichuris globulosa* and tapeworm fragments (presumed anoplocephalids) in seven individuals. Only one calf had an infection with more than one intestinal helminth (tapeworm fragment and *Trichuris globulosa*). The remaining six deer had single species intestinal infections.

No significant age related trends were seen, with the exception of higher intensity of infection of *T. axei* in yearlings relative to other age classes. Assessment of abomasal parasite burden and body condition revealed no significant trends. In calves, statistically non-significant correlation was seen between increased parasite burden and decreased slaughter weight, whilst the opposite was seen in adults with the heaviest adults exhibiting the higher burdens. Given the small sample size the trends that were seen need further investigation. The parasite burden was aggregated with three adult red deer harbouring 75% of the total abomasal parasite count.

**Conclusion:**

This isolated population was parasitised by a reduced subset of gastrointestinal nematodes typical of this cervid across an extensive geographic range in Eurasia. The intensity and abundance of abomasal nematodes was higher in this isolated population than reported in similar studies of red deer populations across Europe.

## Background

Four species of wild cervids are found in Norway: red deer (*Cervus elaphus atlanticus*), moose (*Alces alces*), roe deer (*Capreolus capreolus*) and wild reindeer (*Rangifer tarandus tarandus*). Populations of red deer are primarily found in coastal areas of West and Central Norway, roe deer in East and Central Norway, whereas moose are common in all parts of the country except for West Norway [1,2]. Wild reindeer live in 23 sub-populations in the alpine regions of southern Norway and constitute one of the last populations of wild free-ranging tundra reindeer in Europe, and, therefore, are of great conservation interest. During the past few decades there has been substantial increase in populations of red deer [1,3] coincidental with geographic expansion to the east- and southwards such that these cervids are now present across all municipalities of South and Central Norway. Red deer are also increasingly seen in the wild reindeer areas during summer.

Generally parasite burdens are predicted to increase with density among deer populations [4] and may negatively influence the health status of the animals [5-7]. Increased intensity of parasite infections might, in addition to diminished food resources for hosts, be a contributing factor to the reductions in slaughter weights observed among some of the densely populated red deer areas during the recent years [2]. There is also a concern that the parasite fauna of red deer can be transmitted to sympatric domestic ruminants, such as cattle and sheep as well as other cervid species. There is further concern regarding the potential for parasite transmission from red deer into wild reindeer populations that are also challenged by climate change and environmental constraints. A prerequisite to monitoring the influence of climate change on cervid species and their parasites, as well as detection of host switching events, is development of baseline data on diversity, especially faunal structure for gastrointestinal and abomasal nematodes, among wild red deer from Norway.

**Figure 1:**
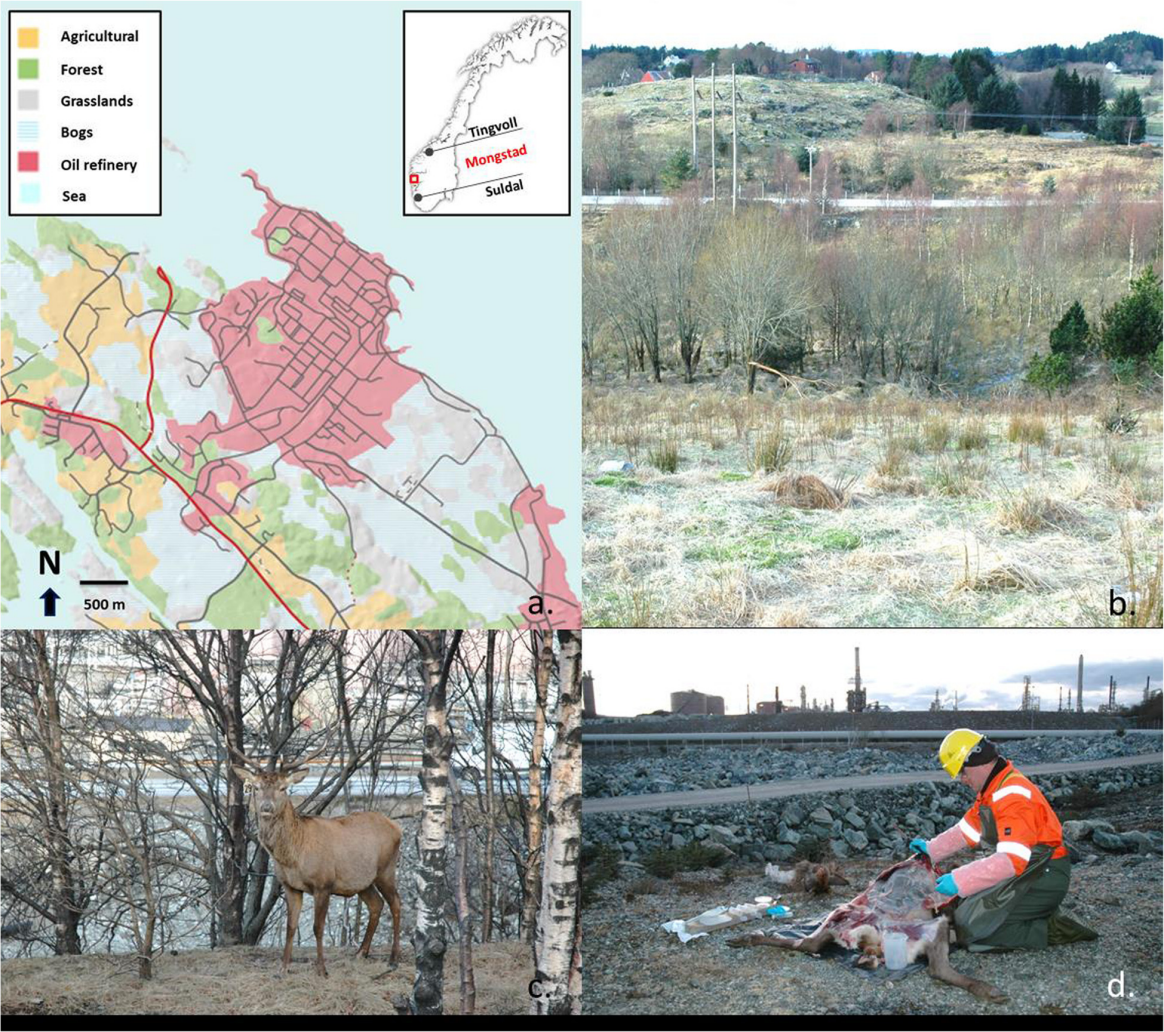
**Map (a) of the Mongstad area with inset of a map of Norway showing Mongstad (red square) as well as Tingvoll and Suldal (black circles); images (b and c) of the area grazed by the red deer, that is enclosed on three sides by a fence and on the fourth by the sea; red deer post-mortem examination in the field (d), with a backdrop of the industrial complex.**

Knowledge regarding biodiversity of gastrointestinal nematodes in wild red deer populations from Fennoscandia is lacking. The sole exception is a Norwegian student thesis [8], describing the gastrointestinal fauna in 25 animals taken by hunters from two municipalities in West Norway in 2005. Although the municipalities studied, Suldal and Tingvoll (Figure 1), are important areas for red deer, considerable numbers of sheep and cattle are also grazed during the summer. Thus, parasite transmission from domestic ruminants may have influenced patterns of helminth diversity. In this regard, red deer were commonly infected with species of Ostertagiinae, although identifications were incomplete, in addition to a broader assemblage of helminths known to occur in other wild cervids and domestic ruminants (Table 1). Parasitological surveys of red deer in continental European countries also identify Ostertagiinae as the predominant components of the abomasal fauna, usually including multiple polymorphic species of *Ostertagia*, *Spiculopteragia* and possibly *Teladorsagia* [9-13]. Polymorphism among males of single species (two or exceptionally three discrete male morphotypes) within this group of parasites has resulted in some inconsistency in reports that document diversity of nematode parasites in ungulates [5,14]. Minor morphs have frequently been reported as discrete species in some studies leading to confusion about host associations and geographic range for some species. Consequently, recognition of polymorphic species and correct morphological and taxonomic identification is vital to provide an accurate picture of diversity [12,15]. Establishing baselines including greater knowledge of the gastrointestinal helminth fauna and accurate species identification of abomasal ostertagiines in red deer is needed to address the potential for cross transmission of parasites among free-ranging and domestic ungulates and can incorporate parasites into management decisions for wild cervid species in Norway.

**Table 1:** **Summary of the results from a gastrointestinal helminth survey in wild red deer (** ***Cervus elaphus*** **) in Norway by Bakka** ***et al.*** [8] **showing the prevalence and parasite counts, including the 95**% **confidence interval (CI), as well as species identified**

**Bakka** ***et al.*** [8]	**N**	**Prevalence [95%CI]**	**Mean counts [95%** **CI]**	**Min-Max (median)**	**Species detected (No. of individuals with each parasite species)**
Abomasal parasites	25	84% [65-94]	820 [523-1116]	0-2300 (700)	*Osteragia* sp. (6)*: *Ostertagia leptospicularis* (4) and *Ostertagia circumcincta* (1)
					*Spiculopteragia sp. (15)*
Small intestinal parasites	25	16% [6-35]	21 [-1-42]	0-200 (0)	*Nematodirus* sp. (4)*: *Nematodirus battus* (2) and *N. filicollis* (1)
					*Capillaria* sp. (1)
					*Moniezia* sp. (3)
Large intestinal parasites	25	72% [52-86]	6 [3-9]	0-20 (3)	*Trichuris sp. (5)*
					*Oesphagostomum venulosum* (14)
					*Chabertia* sp. (1)

*Not identified beyond family level.

The aim of this study was to characterise the gastrointestinal helminth fauna of wild red deer from a dense isolated wild population in an area that for decades has had no other ruminants present, neither domestic nor wild. Also, as a component of diversity, the intensity of infection for helminth parasites was investigated and compared to host body condition, age, and slaughter weight.

## Methods

### Study population and habitat

Mongstad Oil Refinery, owned by Statoil, is situated in coastal western Norway, about 50 km north of Bergen (Figure 1). The refinery was established in the early 1970s and harbours a population of free-ranging red deer. Today, the total fenced area at the refinery is about three square kilometres, but roughly one square kilometre of this area is suitable grazing for deer (grassland, bogs or forest, Figure 1). The population size was estimated to be between 110 and 130 individuals in December 2006, giving a minimum estimated population density of 37 - 43 red deer/km^2^ before culling.

This red deer population is generally considered stationary, due to being enclosed by fencing surrounding the refinery complex. However, single tagged red deer have been registered to leave and return, since there is access to the area via the sea (Figure 1). In 2006, the landowners decided that a cull was necessary to reduce the large population size. Forty-two red deer were culled between February 6^th^ and February 10^th^ 2007, comprising of 14 calves, eight sub-adults and 20 adults. Due to logistical limitations, only 13 animals (three females and ten males) were available for examination. The gastrointestinal tract from each animal was removed and ligated at the time of culling. The tracts were subsequently frozen at -20°C until further analysis could be carried out. The age, sex, body condition and slaughter weight of the animal were recorded. The body condition was assessed based on visible fat reserves subcutaneously and around the heart and kidneys, as good, average, or poor [16].

### Gastrointestinal investigation

Gastrointestinal tracts were thawed at room temperature and then separated into their component regions: abomasum; small intestine; caecum and large intestine. Each component was placed in its own individual 10 l bucket. The abomasum was opened along the greater curvature, the contents emptied into the bucket and the mucous membrane of the abomasum washed thoroughly with cold tap water (≥2 l). The bucket was allowed to sediment for at least thirty minutes before the supernatant was removed to the 2 l level. The remaining 2 l of fluid and sediment were thoroughly mixed prior to removing two 50 ml subsamples for carrying out parasite counts and identification. Abomasal samples were taken from 12 of the 13 culled animals whilst all 13 had their remaining intestinal tracts investigated. The small intestine was visually divided into thirds and the posterior third was removed and discarded. The intestinal contents of the anterior two thirds were flushed into a bucket. The intestine was clipped open longitudinally and the surface of the mucus membrane stripped by placing a finger and thumb to either side of the intestine and pulling the intestine through. Once again the bucket was allowed to stand for at least thirty minutes to sediment. The supernatant was discarded to the 2 l mark. The remaining 2 l of fluid and sediment were thoroughly mixed prior to removing two 50 ml sub-samples for carrying out parasite counts and identification. A minimum 20 g faecal sample was removed from the rectal end of the large intestine and then the intestinal region containing pelleted faeces was removed and discarded. The remaining large intestine and caecum were emptied into a bucket and flushed thoroughly. Both were clipped open and using a finger and thumb together with cold water the mucus membrane was rinsed. The bucket was allowed to sediment for at least an hour before the supernatant was removed to the 3 l level. The remaining 3 l of fluid and sediment were thoroughly mixed prior to removing one 1 l sub-sample for carrying out parasite counts and identification. All the sub-samples were re-frozen at -20°C until further analysis could be carried out. Abomasal digestion to look for arrested larvae was not carried out.

### Nematode count and identification

Parasite counts were done on one 50 ml aliquot from the abomasum (2.5% of abomasal content), one 50 ml aliquot from the small intestine (2.5% of total content) and investigation of 1 l of large intestinal content (33% of total content). The 50 ml fluid was homogenised and a 10 ml subsample transferred to a square line-marked petridish. This was examined under a stereomicroscope at 16x magnification and any nematodes seen were carefully transferred to a sample glass containing 70% ethanol until further analysis could be carried out. This was repeated until the entire 50 ml volume had been examined. The large intestinal contents were first sieved through a 420 μm mesh to remove the largest particles. The fluid was then examined and any parasites seen placed in 70% ethanol in a sample glass. The abomasal and small intestinal nematode counts were multiplied by 40 to obtain a total parasite count. The large intestinal count was multiplied by three to obtain the total count.

Species identification was based on male nematode morphology [14,15,17-20] after mounting in polyvinyl lactophenol (Chemi-Teknikk AS, Oslo, Norway) for two to five minutes. Polymorphism in the Ostertagiinae was accounted for with major and minor morphs recorded separately; counts of major and minor morphotypes representing single species were combined for analysis purposes. A few female parasites were also identified to species level, however degradation of specimens during prolonged storage and repeated freezing and thawing meant that the synlophe and other diagnostic attributes [15] could not be reliably examined for this subset of nematodes. Records reported are validated by representative voucher specimens for helminths collected from red deer in the current study that have been deposited in the US National Parasite Collection, USDA, Agricultural Research Service, Beltsville, Maryland, USA.

### Statistical analyses

JMP statistical software (SAS institute, version 9.0.0) was used to generate and examine summary statistics in the dataset and a significance level of p < 0.05 was selected. Summary statistics were compared among the age groups, body condition and slaughter weight. The 95% confidence interval (CI) was calculated to compare means. The prevalence of the abomasal nematodes in the study population was calculated as well as the proportion of major and minor morphs of each species in addition to the proportion of males to females. Advanced statistical modelling was not carried out given the small size of the dataset.

## Results

The sex of the animals was biased towards males with only three females examined. It was, therefore, not possible to analyse host gender differences in parasitic burden and diversity. The age distribution was five calves, three yearlings and five adults with mean slaughter weight of 22.9 kg, 34.3 kg and 65.3 kg respectively. Assessment of body condition (fat reserves) at slaughter showed that ten animals had average body condition, whilst three had poor body condition: two below average (one calf and one adult) and one emaciated (a yearling).

### Abomasal parasites

Total abomasal counts are shown in Table 2. Species of *Ostertagia*, *Spiculopteragia* and *Trichostrongylus axei* were detected. Ostertagiines were detected in 83% [CI 55 - 95%] of the deer, all of the positive animals had *O. leptospicularis* whilst four also had the minor morph *O. kolchida. Spiculopteragia spiculoptera* (also known as *S. boehmi* according to Drózdz [14]) was detected in 92% [CI 65 - 99%] of the animals and two of the positive animals also had the minor morph *S. mathevossiani. T. axei* was detected in 42%, [CI 19 - 68%] of the deer.

**Table 2:** **Gastrointestinal helminths in 13 red deer (** ***Cervus elaphus*** **) culled from a stationary population around the Mongstad Oil Refinery, Norway, in February 2007**

	**N**	**Prevalence [95%CI]**	**Mean counts [95%** **CI]**	**Min-Max (median)**	**Species detected** ^**a**^ **(No. of individuals with each parasite species)**
Abomasal parasites	12	91.7% [64.6-98.5]	6223 [1262-11185]	0-20480 (1840)	*Ostertagia leptospicularis* (10)/*O. kolchida* (4)
					*Spiculopteragia spiculoptera* (11)/*S. mathevossiani* (2)
					*Trichostrongylus axei* (5)
Small intestinal parasites	13	38.5% [17.7-64.4]	40 [-8-88]	0-280 (0)	*Capillaria bovis* (2)
					Tapeworm fragments (1)
Large intestinal parasites	13	30.8% [12.7-57.6]	1 [0-2]	0-6 (0)	*Cooperia oncophora* (2)
					*Trichuris globulosa* (2)
					*Oesphagostomum venulosum* (2)

^a^Voucher specimens for helminths deposited in the US National Parasite Collection, USDA, Agricultural Research Service, Beltsville, Maryland, USA: *Trichuris globulosa* USNPC 105314; Anoplocephalidae (unidentified cestodes) USNPC 105315; *Spiculopteragia boehmi* (syn. *S. spiculoptera*) USNPC 105316, 105319, 105323; *Ostertagia leptospicularis*/*O. kolchida* USNPC 105317, 105320, 105324; *Capillaria bovis* USNPC 105318; *Trichostrongylus axei* USNPC 105321; *Cooperia oncophora* USNPC 105322.

Aggregation of the parasites was seen with adult hosts harbouring 86% of the total abomasal parasite burden whilst yearlings harboured 8% and calves 6%. Three adult individuals (two males and a female) harboured 75% of the total abomasal parasite burden estimated in this population. The five adult deer examined had significantly higher infection intensities than the five calves, whilst the two yearlings had a lower mean count than adults, although the confidence interval overlapped with that for both calves and adults.

Comparison of body condition estimate and parasite counts showed that two of the three animals with poor body condition, a calf and a yearling, had abomasal parasite counts of 640 and 4 680 respectively whilst the third, an adult, had a count of 18 360 abomasal nematodes. Comparison of the total abomasal parasite burden and slaughter weight by age showed a statistically non-significant trend of decreasing slaughter weight with increasing parasite burden in calves whilst the opposite was true in adults with increasing slaughter weight with increasing parasite burden. However this is based on just five individuals in each age class.

There was a preponderance of female abomasal nematodes compared to male but given that females were not identified we were unable to differentiate counts of the three species detected. The mean ratio of male to female in the complete abomasal parasite count was 1:2.7 (95% CI 1.61 - 3.82). The intensity of infection for male abomasal nematodes (*S. spiculoptera*/*S. mathevossiani*; *O. leptospicularis*/*O. kolchida*), showed no significant differences relative to age of the host with the exception of *T. axei* which was found at significantly higher intensities in yearlings than in the other two age groups. However with only five of 12 individuals infected with this parasite these findings need further investigation. The relative abundance of the three species of abomasal nematodes varied (Table 3). On the whole, the most abundant species was *S. spiculoptera/S. mathevossiani*, followed by *O. leptospicularis/O. kolchida* and the least abundant was *T. axei*. The proportion of major morphs of both *Spiculopteragia* and *Ostertagia* were significantly higher than minor morphs.

**Table 3:** **The different proportions of the three species of abomasal nematodes identified in the abomasal contents of the red deer (** ***Cervus elaphus*** **) from Mongstad (n = 12), based upon morphological identification of all the male nematodes in each sample**

**Parasite species**	**Mean proportion of the different species (males only) [95% CI for the mean]**	**Mean proportion of major morphs [95% CI for the mean]**
*Spiculopteragia spiculoptera/mathevossiani*	64.5 [43.0-86.0]	93.4 [80.0-100.0]
*Ostertagia leptospicularis/kolchida*	26.4 [9.3-43.4]	71.9 [45.0-98.9]
*Trichostrongylus axei*	9.2 [-2.8-20.7]	Not applicable

The 95% confidence interval (CI) for the mean proportions are shown.

### Small and large intestinal parasites

*Capillaria bovis, Cooperia oncophora*, *O. venulosum*, *Trichuris globulosa* and tapeworm fragments (presumed anolpocephalids, possibly a species of *Moniezia*, or *Avitellina*) were recorded in the small intestine of seven individuals (Table 2). Only one of the deer, a calf, had more than one intestinal parasite species with a co-infection of *T. globulosa* and tapeworm fragments.

## Discussion

Red deer from within the enclosure around the Mongstad oil refinery, in 2007, had significantly higher abomasal parasite burdens than free-ranging red deer examined in 2005 from the regions of Tingvoll and Suldal [8]. Further, intensity and abundance exceeded the values reported in similar studies of other red deer populations from localities across Europe [9-12]. High levels of infection in this confined Norwegian population are notable, given that the study was carried out in February in contrast to the traditional hunting season in autumn when parasite burdens would in general be predicted to be higher than those observed during mid-winter [13,21].

A significant difference in abomasal parasite burden was seen between the age classes where greater burdens were associated with increasing age of hosts. Andrews [22] suggested that abomasal nematode loads exceeding 8 000 can be pathogenic and the mean abomasal parasite count in adults in the stationary study population (n = 5) exceeded 12 000. Intensity of infection in our study exceeded levels reported from other, more southern, regions of Europe where red deer have been examined for parasites. Additionally, the prevalence of abomasal parasites in these European studies varied depending on the season, the age class of the host and the parasite species investigated; mean prevalence for the population as a whole generally exceeded 90%. A similarly high prevalence level was seen in the present study indicating that Ostertagiinae remain the most commonly observed gastrointestinal helminths in wild cervidae. The increase in mean abomasal parasite intensity in adults compared to yearlings and calves might be an indication that acquired immunity does not develop with some infections involving species of Ostertagiinae, although improved definition of the adults’ age by year could reveal a more nuanced picture. The exception was *T. axei*, which was recorded at highest levels in the yearlings. Further investigation is needed as to what extent this is a reflection of the high infection pressure in a dense isolated population overcoming partial immunity and to what extent it reflects a lack of immunological stimulation by the parasites.

Two of the three species of abomasal nematodes identified, *S. spiculoptera/S. mathevossiani* and *O. leptospicularis/O. kolchida*, dominated the parasitic picture in the present study with the former being predominant even though both are considered to be characteristic parasites of cervids [12,23]. The more generalist parasite of domestic and wild ruminants, *T. axei* was found at a much lower prevalence in this study population but highlights the fact that wild cervids can act as reservoirs for parasites of relevance to domestic ruminants. The abomasal parasites in red deer from Suldal and Tingvoll were, for the most part, only identified to family level and not species [8]. Investigation of the small and large intestinal species diversity showed no significant differences between the two studies with the majority of the animals in both populations harbouring single species infections even though a number of animals in the study by Bakka *et al.* [8] had up to four concurrent species. Neither of the studies was able to address the reasons for differences in species richness although consideration should be given to cross-infection with cattle and sheep since the population described by Bakka *et al.* [8] shared grazing with domestic ruminants whilst the population in the present study was isolated. For example, this may account for the occurrence of *Nematodirus battus*, *N. filicollis* and *Teladorsagia circumcincta* at Suldal and Tingvoll which are primary helminths of domestic sheep. There were no significant differences in the total parasite count for the small intestine between the two studies although a trend of higher counts was seen in red deer in this study.

Approximately 45 species of gastrointestinal helminths are known in red deer across an extensive range in Eurasia [23]. Diversity reflects the extensive geographic distribution for this cervid in the Palearctic, interactions among sympatric assemblages of free-ranging cervids, and other ungulates and local or landscape effects related to climate [5,24]. Helminths identified in the current study and the survey reported by Bakka *et al.* [8], are a subset of this broader faunal diversity, and there are no new host records documented. In this case, absence of some parasites may indicate environmental limitations on development posed by colder climates characteristic of southern Norway and founder effects on diversity linked to ongoing geographic expansion for red deer, in addition to potential sampling biases. In this regard, and perhaps unexpected, was the complete absence of Nematodirine nematodes at the Mongstad Refinery site, including species of *Nematodirus*, which otherwise are a prominent component of cervid parasite faunas across the Palearctic [23].

Little has been done to explore the gastrointestinal helminth fauna among other wild cervids from Norway, except for one study on abomasal nematodes in wild reindeer [25]. In wild reindeer six different abomasal nematode species were found with *Ostertagia gruehneri/O. arctica* being dominant. The only abomasal nematode reported in wild reindeer shared by red deer in the present study was *T. axei*. However, *S. spiculoptera* has been identified in captive reindeer co-grazed with elk (*Cervus canadensis*) in North America and may be a significant pathogen in this species [26]. Further studies of mixed cervid populations are needed, to understand if the gastrointestinal parasites found in red deer in the present study may establish (or are already shared) by other cervids species in Norway, and more broadly in Fennoscandia [12]. The need for detailed comparative baselines, linked to archived and identified specimens in museum repositories, is indicated by the effects of accelerating climate change and environmental disruption which are influencing the distributions of cervids and other ungulates as hosts, their parasites, and patterns of disease [27].

## Conclusions

The present study was carried out in an isolated wild population of red deer occurring within the core geographic range for this cervid in Norway. Although our sample population was relatively small, results for diversity are expected to be representative for helminth species richness for red deer in this region in the northern extent of the range for *Cervus elaphus*. The intensity and abundance of abomasal nematodes was higher in this isolated population than reported in similar studies of red deer populations across Europe. The species of gastrointestinal helminths recorded were a reduced subset of gastrointestinal nematodes typical of this cervid across an extensive geographic range in Eurasia.
